# An Event-Aware Cluster-Head Rotation Algorithm for Extending Lifetime of Wireless Sensor Network with Smart Nodes

**DOI:** 10.3390/s19194060

**Published:** 2019-09-20

**Authors:** Marcin Lewandowski, Bartłomiej Płaczek

**Affiliations:** Institute of Computer Science, University of Silesia, 41-200 Sosnowiec, Poland

**Keywords:** wireless sensor network, smart node, lifetime, cluster-head rotation, energy consumption, transmission suppression

## Abstract

Smart sensor nodes can process data collected from sensors, make decisions, and recognize relevant events based on the sensed information before sharing it with other nodes. In wireless sensor networks, the smart sensor nodes are usually grouped in clusters for effective cooperation. One sensor node in each cluster must act as a cluster head. The cluster head depletes its energy resources faster than the other nodes. Thus, the cluster-head role must be periodically reassigned (rotated) to different sensor nodes to achieve a long lifetime of wireless sensor network. This paper introduces a method for extending the lifetime of the wireless sensor networks with smart nodes. The proposed method combines a new algorithm for rotating the cluster-head role among sensor nodes with suppression of unnecessary data transmissions. It enables effective control of the cluster-head rotation based on expected energy consumption of sensor nodes. The energy consumption is estimated using a lightweight model, which takes into account transmission probabilities. This method was implemented in a prototype of wireless sensor network. During experimental evaluation of the new method, detailed measurements of lifetime and energy consumption were conducted for a real wireless sensor network. Results of these realistic experiments have revealed that the lifetime of the sensor network is extended when using the proposed method in comparison with state-of-the-art cluster-head rotation algorithms.

## 1. Introduction

Recent developments in smart sensor technology have opened new perspectives for advanced applications of wireless sensor networks (WSNs) in many domains including healthcare, automation, infrastructure, and environment. The smart sensor nodes in WSN enable complex processing and analysis of sensed data with increased computational power [1,2]. WSNs have become an important part of 5G mobile technology that open new perspectives for advanced applications of smart sensors in future Internet of Things (IoT) applications [3,4].

Typical WSNs are composed of nodes that sense their environment by using built-in sensors and transmit the sensor readings to a sink node [5]. In contrast, smart sensor nodes have an additional ability to process the collected data, make decisions, and recognize relevant events based on the sensed information before sharing it with other nodes [6]. Instead of transmitting raw data readings, the smart sensor nodes report the detected events. Moreover, to recognize the important events in larger regions, many smart nodes must cooperate. It means that the sensor nodes from a given region of interest are grouped into cluster and exchange sensed information to make a common decision regarding occurrence of an event [7,8]. In this case, the individual sensor nodes assess partial detection results and report them to one selected node, which acts as a cluster head (CH). One CH node is selected for each cluster. The CH node combines the collected partial results to recognize events in the region of interest. Finally, the information about events is reported by CH node to the sink.

The key issue in applications of wireless sensor networks is the limited lifetime of battery-powered sensor nodes. Thus, network management methods are necessary to enable effective use of the energy resources of sensor nodes [9,10].

In this study, the term lifetime refers to the time interval of the WSN operation before death of the first node. It should be noted that the uninterrupted operation of all sensor nodes in WSN is crucial for many applications where the feedback from the network must be reliable [11].

This paper introduces a method for extending lifetime of the above-mentioned wireless sensor networks with smart nodes. The proposed method combines a new algorithm for rotating the CH role among sensor nodes with suppression of unnecessary data transmissions.

Data transmission is the most energy expensive operation for energy constrained wireless sensor nodes. According to the suppression approach, consumption of battery power in sensor nodes is reduced by transmitting the data only if it is necessary [12]. In the considered scenario, a smart sensor node, which did not detect any relevant event, can skip transmission to CH node to save the energy. In general, the transmissions of partial results to CH can be suppressed if the event detection results that would be obtained by the CH with and without these partial data are the same.

To prolong the lifetime of wireless sensor network, a balance must be achieved between energy consumption of all sensor nodes. CH node consumes more battery energy than the remaining sensor nodes. The reason behind unequal energy consumption is that CH node must remain active most of the time, while other nodes are active only when processing and transmitting data. During inactive time, the sensor nodes are switched to sleep mode for saving energy. Thus, the CH node depletes its energy resources faster than the other nodes, which leads to reduced network lifetime. The balance of energy consumption can be restored by periodically reassigning (rotating) the CH role to different sensor nodes [13].

It should be also noted here that the energy balance is significantly disrupted by the mechanism of transmission suppression. The smart sensor nodes that detect a higher number of important events must perform the data transmissions more frequently and consume a larger amount of energy.

The main contribution of this paper is a new CH rotation algorithm, which takes into account individual energy savings achieved by the smart sensor nodes that suppress the unnecessary data transmissions. The proposed CH rotation algorithm was implemented in a prototype of wireless sensor network. During experimental evaluation of the new algorithm, detailed measurements of real energy consumption and network lifetime were conducted. Results of these experiments have revealed that lifetime of the prototype sensor network is extended when using the proposed method in comparison with state-of-the-art CH rotation algorithms.

The paper is organized as follows. Related works are surveyed in Section 2 and Section 3 includes presentation of the proposed algorithm, which enables extending lifetime of the sensor network. Experiments and their results are described in Section 4. Finally, conclusions are given in Section 5.

## 2. Related Works and Contribution

In the literature several methods have been proposed to extend lifetime of WSNs by properly managing CH nodes. This section briefly reviews the state-of-the-art methods for CH selection in WSNs and discusses the main contribution of the paper.

The basic method for organizing data transfers in WSN is direct transmission [14]. This method assumes that all sensor nodes have the same role and their task is to send all sensors readings to a base station (sink). In this case, the CH nodes are not present. It should be noted that the sensor nodes consume energy mainly when they are accessing the communication channel and transmitting data. Thus, the transmission of all sensed data to base station results in high energy consumption and reduced lifetime of sensor nodes. Moreover, scalability of this method is low as the base station must collect and process large amounts of data from all sensor nodes. If workload of the base station is too high, the sensor data may be lost. The complexity of direct transmission protocol is negligible, and its implementation is simple. However, the low energy efficiency of this protocol makes it not suitable for most applications.

Limitations of the direct transmission have motivated the development of more sophisticated data collection methods that divide the WSN into clusters. These methods reduce the number of data transmissions and save energy of sensor nodes. The sensor nodes in a given cluster send information to CH node, which aggregates the data and transmits them further to the sink or to another CH. However, as already mentioned in Section 1 the CH role has to be rotated among sensor nodes to balance their energy consumption and avoid fast energy depletion of the nodes that takes CH role for a longer time.

The available CH rotation algorithms can be divided into two categories. The first category includes time-driven algorithms. According to these algorithms the sensor nodes take the role of CH for a predetermined time period. The second group consists of energy-driven algorithms that take into account residual energy of sensor nodes and change CH node when a predetermined portion of the energy is consumed.

### 2.1. Time-Driven Cluster-Head Rotation Methods

A distributed algorithm for organizing sensor nodes into clusters was introduced in the hierarchical routing protocol called LEACH (Low-Energy Adaptive Clustering Hierarchy) [15]. According to this protocol, the CH role is randomly rotated among the sensor nodes to not drain the battery of a single sensor node. New CH node is selected after a certain time (round), which is determined a priori. The CH node creates TDMA (Time Division Multiple Access) schedule. Thus, the sensor nodes send their data in time slots allocated by the CH node. The CH node collects data and sends them to the base station. Operation of the LEACH protocol is divided into rounds and each round consists of two phases: set-up phase and steady phase. In set-up phase the new CH is selected and sends an advertisement, which includes information about its role. In the steady phase data transmission begins and sensor nodes send their data to the CH node according to allocated TDMA slots. LEACH allows the CH nodes to perform local aggregation of data in each cluster to reduce the amount of data transmitted to the base station.

In centralized version of the LEACH protocol (LEACH-C) [16,17] each node sends information about its current location and residual energy level to the sink, which uses a centralized clustering algorithm to select CH nodes. The sink computes average node energy, and the sensor nodes with energy below this average cannot be selected as CH for the current round. This method aims at evenly distributing the cluster-head nodes throughout the network. However, the centralized approach requires additional data transmissions form sensor nodes and involves a complex clustering procedure, which must be performed by the sink.

LEACH-D [18] is a modification of the original LEACH protocol, which assumes that after initialization of the network, the sensor node with the shortest distance to center of the cluster becomes CH. After each round, the closest neighbor of current CH node is selected as a new CH, provided that its residual energy is more than a threshold. Otherwise, the same condition is verified for the next closest neighbor. In case when there is no neighboring sensor node with residual energy higher than a threshold, the sensor node with minimum distance to the current CH is selected as the new CH.

Fixed clusters of sensor nodes are considered in LEACH-F [19]. In this approach the clusters are formed once, using the centralized clustering algorithm developed for LEACH-C. The sensor nodes within a cluster are numbered and the CH position rotates among these nodes according to the round-robin method. It means that the first sensor node becomes CH for the first round; the second node is CH in the second round, and so forth. This protocol eliminates the communication overhead of the cluster formation procedure at the beginning of each round. However, it is not suitable for dynamic networks.

In HEED (Hybrid Energy-Efficient Distributed Clustering) protocol [20] the new CH nodes are also selected after a predetermined time, corresponding to duration of one round, which is similar to the methods discussed above. However, in case of HEED the CHs are selected by taking into account the amount of residual energy. For each sensor node the probability of becoming CH is proportional to its battery level. The distributed CH selection procedure requires only local information about neighboring sensor nodes, but involves considerable energy expenditure to periodically rebuild clusters.

Another time-driven method is ANTCLUST [21]. In this method the CH nodes are selected at the beginning of each round, as in previous approaches. To improve the network lifetime ANTCLUST selects the new CHs according to battery level and distances to neighboring sensor nodes. A specific feature of this method is the application of the clustering algorithm based on a model of the chemical recognition system of ants.

Stable Election Protocol (SEP) [22] was intended for heterogeneous sensor networks, where sensor nodes have different initial levels of energy. In SEP new CH nodes are randomly selected at the beginning of each round, just like in LEACH. An advantage of this protocol is that sensor nodes with higher energy become CHs more often than the nodes with lower energy resources. This approach prolongs the time interval before the death of the first sensor node.

A modified Stable Election Protocol, named Prolong-SEP (P-SEP) was presented in [23] to extend the lifetime of fog-supported sensor networks by maintaining balanced energy consumption. P-SEP enables uniform nodes distribution and introduces two energy levels for normal and advanced nodes. The nodes with energy level above the threshold are nominated as CH candidates and then the CHs are randomly selected from the candidates, by taking into account their distance to fog nodes. It should be noted that in both SEP and P-SEP algorithm, the selection of CH node is an element of a complex clustering procedure. Thus, those methods can be ineffective in frequent rotating CH role for predetermined clusters, where changes of cluster membership are not required.

In [24] a protocol was presented called HEER (Hamilton Energy-Efficient Routing Protocol). HEER uses greedy algorithm to construct Hamilton path between sensor nodes in each cluster. This path is used for data transmission purpose. Sensor nodes on the path take the role of CH successively in turns. As for other time-driven methods, the change of CH occurs after operating for a predetermined round time.

### 2.2. Energy-Driven Cluster-Head Rotation Methods

Energy-distance aware clustering method (EDAC) [25] elects CHs based on two parameters: the residual energy of sensor nodes and the energy expended for transmitting data from sensor nodes to potential CH. These two parameters are combined in a metric, which quantifies how good a sensor node would be as a CH. Using the metric evaluated for all sensor nodes in a cluster, each node decides whether it will be the new CH. Selection of new CH is made when the residual energy of current CH drops below a predetermined threshold.

In case of energy-distance aware clustering scheme for wireless sensor networks (E-DACS) [26] the CH is selected for each cluster depending on its residual energy, distance from other sensor nodes in cluster, and distance to sink. New CH selection is executed periodically, once energy level of current CH becomes less than the energy of any other sensor node in its cluster.

Energy-driven cluster-head rotation (EDCR) is an algorithm where the selection of CHs is based on relative residual energy level of sensor nodes in a given cluster. The sensor node with the highest energy level becomes the CH. A dynamic threshold is used to trigger the CH rotation process. The threshold is calculated as P·EC, where 0<P<1 is a constant parameter, and EC is the residual node energy, measured when the node is selected to be CH. In this method, the selection of new CH takes place when the residual energy of CH drops below the threshold value. The lower is the residual energy of sensor nodes, the more often the change of CH node takes place. This rotation strategy was intended to balance the energy consumption in sensor network. An extension of the EDCR algorithm is the EDCR-MH [27], which has been adapted to the requirements of multi-hop networks.

For sensor networks with energy harvesting capabilities, a method was proposed in [28] to select CH nodes by taking into account predicted rates of energy harvesting. That method uses back propagating neural network for the prediction purposes. The sensor nodes with more available energy and closer to the center of a cluster are more likely selected to become CHs. As a result, the node energy consumption is balanced among the cluster.

### 2.3. Our Contribution

The above-discussed state-of-the-art CH rotation methods have important drawbacks that limit their effectiveness in applications to WSNs with smart nodes. First of all, the existing methods are based on an assumption that all sensor nodes in a cluster transmit their sensed data with the same, constant frequency. The possibility of suppressing data transmissions by the smart sensor nodes is not taken into account in those methods. It should be kept in mind that the considered smart nodes perform transmission when recognizing an important event. Each node can detect different number of events and the frequency of events can change in time. It means that the energy consumption of smart node changes dynamically and may differ significantly between nodes. Thus, in case when the smart nodes in a cluster suppress unnecessary transmissions, the existing methods can fail in achieving the balance of energy consumption for all nodes, which leads to reduced network lifetime.

This paper introduces a new CH rotation method, which takes into account probabilities of detecting events and transmitting data by smart sensor nodes. According to this method, the CH node estimates the probabilities of data transmission for all cluster members. Based on the estimated transmission probabilities, using a lightweight energy consumption model, the CH node decides when the change of CH must take place. The determined time of CH change ensures equal energy consumption for all nodes in the cluster. This leads to maximization of the network lifetime, which is defined as the time to death of the first sensor node.

Another drawback of the state-of-the-art methods is that they require parameters calibration for each application and each deployment of sensor network. For instance, the most important parameters that have significant impact on effectiveness of those methods, and need to be carefully calibrated, are the time duration of one round (for time-driven approaches) and the energy threshold (for energy-driven algorithms).

In the proposed method, it is necessary to determine parameters of an energy consumption model. These parameters are related to hardware solutions used to build sensor nodes, and can be determined in advance for a given node architecture. Therefore, the experimental calibration of the parameters after installation of the sensor network is not performed.

The contribution of this work also includes experimental evaluation of the CH rotation methods with use of real WSN. Comparison of the proposed method with time-driven and energy-driven approaches was conducted for physical prototypes of smart sensor nodes. Results of this study include measurements of WSN lifetime in real-world conditions. In contrast, the results presented in related works were obtained using simulation software.

## 3. Proposed Method

### 3.1. Cluster-Head Rotation

The objective of the proposed method is to prolong the lifetime of WSN by appropriately rotating the CH role among smart sensor nodes. It should be kept in mind that the considered WSN lifetime corresponds to time interval before death of the first sensor node.

**Figure 1:**
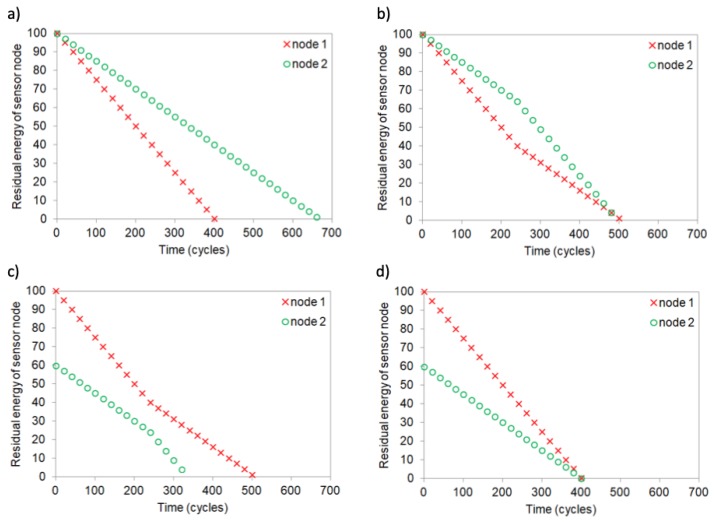
Changes of residual energy in WSN for different CH rotation scenarios: (**a**) the same initial energy levels without CH change, (**b**) the same initial energy levels with CH change, (**c**) different initial energy levels with CH change, (**d**) different initial energy levels without CH change.

The problem of achieving maximum WSN lifetime is illustrated in Figure 1 for a network composed of two sensor nodes. The examples shown in Figure 1a,b) assume that both nodes have the same initial energy level (energy = 100 for time = 0). In the example from Figure 1a)a static assignment of the CH role is considered, i.e., node 1 takes the CH role for the entire analyzed period. Thus, the residual energy of node 1 decreases faster than the energy of node 2. As a result, node 1 dies after 400 cycles, while node 2 has still 40 units of energy (it should be noted that time in Figure 1 is expressed in cycles of sensor node operations). The lifetime of WSN in this example equals 400 cycles.

The second example (Figure 1b) shows that the lifetime of WSN can be extended by changing the sensor node, which takes the CH role. In this example, node 1 takes the CH role for cycles 0–249, and then the CH role is performed by node 2. Both sensor nodes deplete their energy at the same time step, after 500 cycles. It means that lifetime of the WSN is prolonged to 500 cycles. The lifetime of 500 cycles is the maximum time for the considered WSN example. It should be noted that the maximum lifetime is achieved when both sensor nodes die at the same time step.

Lifetime of the WSN for different initial energy levels of sensor nodes is analyzed in Figure 1c,d). In these examples the initial energy equals 100 units for sensor node 1 and 60 units for sensor node 2. Figure 1d) shows that without CH role rotation (node 1 is CH) both nodes die simultaneously after 400 cycles of the WSN operation. Thus, for the above-mentioned levels of initial energy the maximum WSN lifetime is equal to 400 cycles. In Figure 1c) the change of CH node after 250 cycles results in decreased WSN lifetime, as node 2 depletes its energy approximately at cycle 330.

When analyzing the examples from Figure 1b,d) it can be observed that the WSN lifetime is maximized if both nodes die at the same time. This observation is also confirmed by conclusions of the related works, e.g., [23,28], where more complex scenarios were considered than those discussed above. Therefore, the general rule can be formulated that energy consumption of sensor nodes has to be balanced to prolong the WSN lifetime.

Expected lifetime of sensor node *i* can be evaluated as $EI_{i}$/$e_{i}$, where $EI_{i}$ denotes initial energy level of *i*-th node and $e_{i}$ is average energy consumed per one cycle of WSN operation by sensor node *i*. For instance, in Figure 1b) $e_{1}$ = $e_{2}$ = 0.20, while in case of Figure 1d) $e_{1}$ = 0.25 and $e_{2}$ = 0.15. Thus, the following condition must be satisfied to achieve the maximum lifetime:

(1) $$\frac{EI_{1}}{e_{1}}=\frac{EI_{2}}{e_{2}},$$

The average energy consumption ($e_{i}$) of *i*-th sensor node in time period *T* depends on the fraction of time *T* in which sensor node *i* takes the CH role. Let us denote this fraction of time as $t_{i}$ then the average energy consumption can be expressed as follows:

(2) $$e_{i}=EH\;\cdot \;t_{i}+EM\;\cdot \;\left(1-t_{i}\right),$$

where $t_{i}\in$ [0, 1], EH and EM denote energy consumed during one cycle by a sensor node which takes cluster-head role or cluster member role, respectively. In the considered WSN with smart nodes the energy consumption of cluster member takes different values depending on event detection. A larger amount of energy is consumed if at a given cycle the sensor node detects an important event and reports it to the CH node. Lower energy consumption is encountered when the event is not detected, and the sensor node suppresses data transmission. Therefore, in the proposed approach energy EM is estimated by taking into account the probability of event occurrence for i-th sensor node $p_{i}$:

(3) $$EM=EMH\;\cdot \;p_{i}+EML\;\cdot \;\left(1-p_{i}\right),$$

where EMH denotes energy consumed by the sensor node (cluster member) during a cycle when an event is detected and data transmission is performed, EML is energy consumption of sensor node during a cycle when no event is detected and transmission is suppressed. The event occurrence probability $p_{i}$ is evaluated based on historical data.

The fractions of time when particular sensor nodes take the CH role ($t_{i}$) must be carefully selected to maximize the network lifetime. According to the proposed method, the time fractions $t_{i}$ are determined by solving the following system of linear equations to ensure the same expected lifetime for all sensor nodes:

(4) $$\left\{\begin{array}{c} \frac{EI_{i}}{e_{i}}=\frac{EI_{i+1}}{e_{i+1}},i=1,...,n-1\\ \sum_{i=1}^{n}t_{i}=1 \end{array}\right.,$$

where *n* is the number of sensor nodes in a WSN cluster, and the remaining symbols are defined above. The equation system (4) can be solved by CH node or base station (sink node). It should be noted that various algorithms for solving the system of linear equations are available in the literature, e.g., [29,30,31].

After calculating time fraction $t_{i}$, the length of time when sensor node *i* has to take the CH role is determined as $TCH_{i}=T\;\cdot \;t_{i}$, where *T* denotes time of one round, i.e., a predetermined time during which each sensor node in the cluster acts as CH.

### 3.2. Algorithms for Sensor Nodes

Detailed implementation of the above-discussed CH rotation rule is presented in form of algorithms executed by sensor nodes.

The smart sensor nodes in WSN execute the operations presented by the pseudo codes in Algorithms 1–4. The operations are repeated in regular time intervals (steps). During normal operation the non-CH node (cluster member) reads data from its sensors, detects events based on the collected sensor readings and, if an important event is detected, transmits the detection results to CH node (see Algorithm 1). For energy saving, the sensor node wakes its communication module up only when the data transmission is necessary. In case when the initial energy of sensor nodes is not known in advance, the cluster member can include information about its residual energy in the first data frame, which is send after selection of new CH. As shown in Algorithm 2, the normal operation of CH node consists of collecting sensor readings, detecting events, aggregating results received from cluster members, and sending the aggregated results of event detection to sink. Once the last step of the normal operation period is finished, all sensor nodes in WSN switch to executing the CH selection algorithm (Algorithm 3).

**Algorithm 1** Node operating in cluster member mode
1:step = step + 12:read data from sensors3:detect event4:**if** important event detected **then**5:    wake communication module up6:    send event detection result to cluster head7:
**end if**
8:**if** step = last step **then**9:    **if** communication module sleeps **then**10:        wake communication module up11:    **end if**12:    run cluster-head selection algorithm13:
**end if**
14:put communication module to sleep (sleep time)


**Algorithm 2** Node operating in cluster-head mode
1:step = step + 12:duty time = duty time + 13:read data from sensors4:detect event5:wake communication module up6:broadcast sync frame7:receive event detection results from cluster members (wait time)8:aggregate event detection results9:send aggregated results to sink node10:update event counter11:**if** step = last step **then**12:    run cluster-head selection algorithm13:
**end if**
14:put communication module to sleep (sleep time)


According to the proposed CH selection approach, a new CH node is selected if the current CH node performs this role not shorter than the time $TCH_{i}$, which is calculated using the method presented in Section (see line 4 in Algorithm 3). This approach requires the probabilities of event occurrence ($p_{i}$) to be evaluated for all sensor nodes in the cluster. To this end the CH node holds an event counter that indicates how many times given sensor node has reported an event. The probability $p_{i}$ is estimated as a quotient of the value recorded in event counter divided by the duty time. It should be noted that the variable called duty time in Algorithms 2 and 3 informs how long the current CH fulfils the CH role. If the aforementioned condition of CH change is satisfied then the next sensor node from a predetermined sequence is selected to act as CH. The sequence is established during initialization of the WSN and corresponds to the order in which the sensor nodes have joined the cluster. In case when the CH change condition is not met, the current CH node stays in its role.

For large clusters with many sensor nodes, the computations of $TCH_{i}$ that require solving the system of linear equations (4), can lead to significant energy expenditure and computation time, when performed by the CH node with limited resources. Thus, an optional procedure was proposed where the system of equations is solved by the sink (base station), which is powered from the mains and has greater computational power than CH node. According to this procedure the CH node sends a request to the sink with estimated probabilities of event occurrence ($p_{i}$) and receives the $TCH_{i}$ time. It should be also noted that the equation system can be effectively solved by using artificial neural networks [32] or approximate algorithms [33]. Moreover, an approximate solution is sufficient for the proposed CH rotation method, as the $TCH_{i}$ time must be determined with precision that corresponds to the time interval between two successive CH selections. At the time of CH selection procedure, the cluster members listen to sync frame, which is broadcasted by the CH node. The sync frame includes address of the new selected CH. If a cluster member receives the sync frame with CH address equal to its own address then it switches to act as CH node. Another basic function of the sync frame is time synchronization of all nodes in the cluster. The sensor nodes are synchronized by resetting their timers on reception of the sync frame.

**Algorithm 3** Cluster-head selection
1:**if** node role = cluster head **then**2:    determine probabilities $p_{i}$ based on event counter3:    determine $TCH_{i}$ using method presented in Section 3.24:    **if** duty time >= $TCH_{i}$ **then**5:        CH address = next address in sequence6:        node role = cluster member7:    **end if**8:    broadcast sync frame9:    put communication module to sleep (sleep time)10:
**else**
11:    listen to sync frame (wait time)12:    **if** sync frame received **then**13:        **if** CH address = own address **then**14:           node role = cluster head15:           duty time = 016:        **end if**17:        put communication module to sleep (sleep time)18:    **else**19:        begin node initialization procedure20:    **end if**21:
**end if**
22:step = 0


The algorithm of sensor node initialization (Algorithm 4) is executed every time a sensor node starts to operate as well as when CH node is no longer present, e.g., due to malfunction or battery depletion. The initialized sensor node waits for sync frame. If the sync frame is received, the sensor node joins the WSN cluster as cluster member. In opposite situation, when sync frame is not received, the node takes the CH role. It should be noted that the sync frame includes information about current step of cluster operation. The CH node broadcasts the sync frame periodically, as shown in Algorithm 2 (line 4). Thus, a sensor node can join the cluster at any time, e.g., after its recovery.

**Figure 2:**
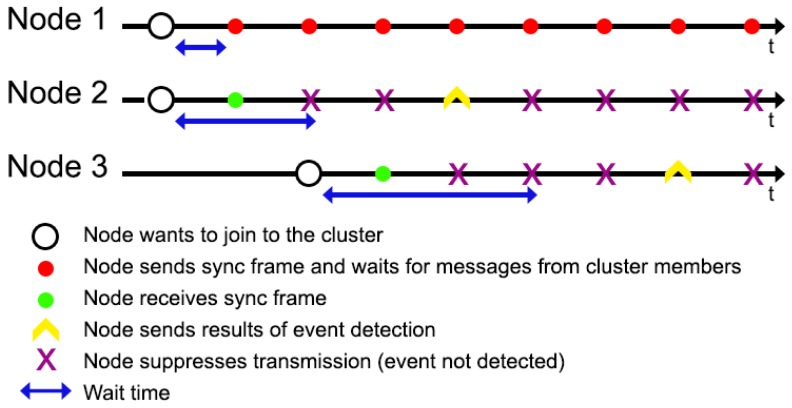
Initialization process for 3 nodes in WSN cluster.

During initialization, the wait time, i.e., time when sensor node waits for the sync frame, is different for each node. The wait time (sync wait time) depends on address of the node (see line 2 in Algorithm 4). This prevents the situation when many neighboring sensor nodes take the CH role at the same time. An example of WSN cluster initialization is presented in Figure 2. In this example sensor nodes 1 and 2 are initialized at the same time and want to join the WSN cluster. Initially, the CH node is not present, thus node 1 does not receive sync frame during its wait time, and becomes the CH. As node 1 takes the CH role, it starts broadcasting sync frames. One of such frames is received by node 2, which then join the cluster as cluster member. Similarly, the 3rd sensor node, which starts its operation later than the other nodes, acts as cluster member after receiving the sync frame.

The most computationally complex operation in the proposed approach is to solve the system of linear equations in Algorithm 3 (line 3). In this work, this operation was performed by using the exact algorithm presented in [31], with complexity of $O(N^{3})$, where *N* is number of cluster members. As explained above, the computational complexity can be reduced by implementing heuristic or approximate algorithms to solve the equation system. Moreover, this computationally demanding operation can be effectively performed by the base station (sink) for larger clusters. The remaining operations of the proposed algorithms have linear complexity (O(N)).

**Algorithm 4** Initialization of sensor node
1:wake communication module up2:sync wait time = delay * address3:listen to sync frame (sync wait time)4:**if** sync frame received **then**5:    role = cluster member6:    step = step determined in sync frame7:
**else**
8:    role = cluster head9:    step = 010:    duty time = 011:
**end if**
12:put communication module to sleep (sleepTime)


## 4. Experiments

The objective of the experiments was to measure the energy consumption of sensor nodes and to estimate the lifetime of WSN. During experiments the lifetime of WSN with smart nodes was compared for the proposed CH rotation method and the state-of-the-art approaches, including time-driven and energy-driven CH rotation algorithms.

### 4.1. Experimental Testbed

The WSN built for research purposes consists of four smart sensor nodes. Three of the sensor nodes can act as cluster head or cluster member. The fourth node is used as sink. Each node of the WSN contains a microcontroller, a communication module and an analogue light sensor ALS-PT19. The measurement of energy consumption is performed by using LTC4150 Coulomb counter [34], which is an external module of the microcontroller.

The Coulomb counter is useful in measuring the depletion of battery energy in wireless devices. It enables continuous current measurement in situation where long-term observation is required. The measuring process is based on differential measurement over a shunt, i.e., a resistor that has a small amount of resistance and is connected in series. The Coulomb counter detects potential difference (voltage drop) across the resistor and determines current flowing through the circuit using Ohm’s law. Due to parameters of the internal resistor and the maximum energy consumption of the sensor node, the smallest amount of energy to be measured is 26 µAh. The LTC4150 module generate impulse on an output pin each time 0.1707 mAh is absorbed by the sensor node. These impulses are counted by an additional microcontroller to evaluate the energy consumption over a long period of time. It was assumed that all sensor nodes have the same battery capacity of 700 mAh.

When designing smart sensor nodes, two families of microcontrollers were taken into account: ARM and AVR. Both can switch off unused functional blocks, but differ in the approach to energy saving. The microcontrollers of AVR family (ATTiny, ATMega) by default have all modules such as ADC, $I^{2}C$, SPI enabled. Different approach is used in ARM systems. Here, initially all modules are switched off. It is also possible to turn off the power supply to individual input/output ports, which in the case of the aforementioned AVR family of microcontrollers is not possible. In this research the ARM microcontroller was used (STM32F103), which is clocked at 72 MHz frequency and offers greater accuracy of timers, enabling better synchronization of sensor nodes, while maintaining compact dimensions of the device.

**Figure 3:**
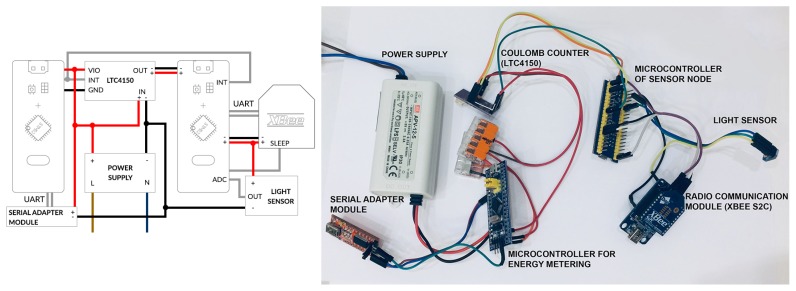
Part of experimental testbed for one sensor node.

The wireless communication module installed in sensor nodes is based on ZigBee technology (xBee S2C), which provides wireless end-point connectivity to devices. These modules use DigiMesh firmware developed by Digi. DigiMesh simplifies network configuration and increases reliability in conditions where devices required for network operation may fail. Moreover, this technology is easy to use. The xBee modules support multiple network topologies such as point to point, point to multi-point, mesh, and cluster tree. The system includes an implemented data retransmission mechanism. The electrical diagram of the test environment for single sensor node was shown in Figure 3.

As shown in Figure 3. an additional node (microcontroller without wireless communication module) is included in the experimental testbed to measure the energy consumption for all sensor nodes. The measurements collected by the additional nodes are not available for the sensor nodes and are used only for evaluation of the WSN lifetime. Thus, the sensor nodes cannot use these measurements for making decisions about CH rotation.

Initial research was conducted to verify the possibility of evaluating both the energy consumption and the WSN lifetime with use of sensor nodes powered from batteries. However, when repeating the measurement with the same settings, significantly different results were obtained. This problem occurs due to the fact that the batteries are never charged exactly in the same level. To solve this problem a power supply unit, Mean Well APV-12-5 [35], was used during experiments. The lifetime of WSN was determined for each analyzed scenario by the additional node, which performs measurements of energy consumption. Death of sensor node was detected each time the sensor node has consumed a predetermined amount of energy (3500 mWh). This approach has ensured repeatability of the conducted experiments.

**Figure 4:**
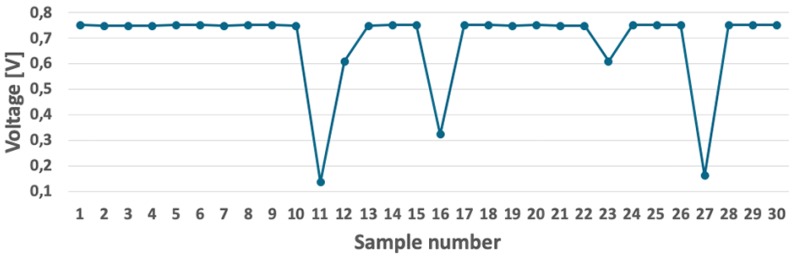
Example of light sensor readings collected during changes in light level.

The considered WSN was designed for indoor detection of moving objects, e.g., people passing through a corridor in a building. Therefore, each sensor node was equipped with a light sensor [36], which converts the lighting level into a proportional value of the voltage at its output. This voltage is then measured through one of the ADC (Analogue-to-Digital Converter) channels of the microcontroller. An example of lighting level measurement is shown in Figure 4. It should be noted that the designed sensor nodes recognize important events as significant changes in the voltage level at the light sensor output. To be detected as event, the registered changes must be above a threshold, which is determined dynamically by using a moving average filter [37]. Determining the threshold in this way guarantees correct detection of objects in various lighting conditions. The moving average filter is used with time window of 100 samples.

The smart sensor nodes were programmed to perform the algorithms presented in Section 3.2. In this implementation, one step of the WSN operation corresponds to 0.5 s. It means that the sensor data are collected, event occurrence is verified, and result is reported to the sink two times per second. This enables detection of fast-moving objects.

For detailed evaluation of the proposed CH rotation algorithm it was necessary to conduct experiments with various probabilities of event detection by particular sensor nodes. It would be difficult to control the light intensity to achieve a desired probability of event detection. Therefore, a function was programmed, which allows the sensor nodes to randomly generate the events with a given probability. lAs a result, the smart sensor node performs transmission when an event is randomly generated. If no event is generated then the data transmission is suppressed. The simple suppression procedure does not require any additional parameters. This approach has significantly facilitated the experimental evaluation.

**Figure 5:**
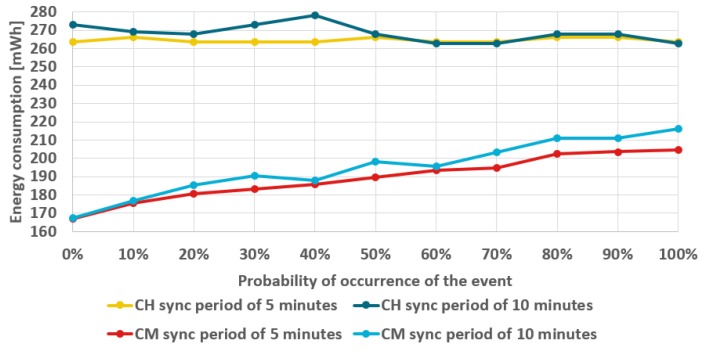
Impact of the synchronization period on the energy consumption of sensor nodes.

During tests of the elaborated WSN it was observed that increased energy consumption is caused by retransmissions that occurs in case of incorrectly synchronized sensor nodes. It should be noted that the cluster members send data to CH node according to TDMA schedule and their timers need synchronization to avoid transmission errors due to collisions. Moreover, if cluster member is not correctly synchronized their messages can be not received by the CH node. In such situations, when frame transmission is not finished successfully, the xBee modules perform retransmissions. The impact of synchronization period on energy consumption by sensor node is illustrated in Figure 5. The results presented in Figure 5 were obtained for two synchronization periods: 5 min and 10 min. In the first case the CH node consumed 265 mWh of energy on average, while cluster member used 189 mWh. After extending the synchronization period to 10 min, the average energy consumption increased to 268 mWh for CH node, and to 195 mWh for cluster member. The sensor nodes consume less energy when the synchronization is performed every 5 min since in this case fewer retransmissions are necessary. The number of retransmissions is reduced as the sensor nodes are better synchronized and send their data in the TDMA slots allocated to them, exactly when the CH is waiting for the data. In case when the synchronization is performed every 10 min, the number of transmissions executed out of the specified time slots increases at the last phase of the synchronization period. Such transmissions cannot be completed correctly and the data are retransmitted at the next opportunity. Based on these results, a short synchronization period was used in further experiments.

Additionally, Figure 5 shows changes of energy consumption as a function of the probability of event occurrence. When analyzing these results, it should be noted that for cluster member the energy consumption increases with the probability of event occurrence. The reason behind this dependency is that the smart sensor nodes suppress transmissions to CH if no important event is detected. Thus, if the event probability is higher, the cluster member must perform the transmission more frequently. In contrast, CH member reports the aggregated results to the sink at each step of the WSN operation and its energy consumption does not change significantly with the event probability.

The network lifetime can be determined with the different assumptions described in the previous section. Due to the nature of the detected data, which is implemented in a relatively small area, the network lifetime is defined in this study as the time from the start of all nodes to the discharge of one of them. This is due to the exponential drop in detection quality after any of the nodes are switched off. Battery discharge is indicated by the blinking LED that was attached to the microcontroller module.

### 4.2. Results and Discussion

Tests of the CH rotation algorithms were divided into two parts. The first part is devoted to the situation where the probability of event occurrence is constant in time. In this case, the event probabilities for sensor nodes 1–3 were set to 0.2, 0.3, and 0.5, respectively. The second part of tests deals with a more realistic case in which the event occurrence probability varies with time. For instance, the probability of detecting person in an office building is high during working hours and low throughout the remaining part of the day. During experiments the probabilities were changed every 5 min in cycles of 15 min. In case of the first sensor node the probability for three successive 5-min intervals was 0.2, 0.2, and 0.3. In a similar way, the probabilities of 0.6, 0.7, and 0.9 were assigned to sensor node 2. Finally, sensor node 3 was detecting the events with probabilities of 0.1, 0.4, and 0.1.

**Figure 6:**
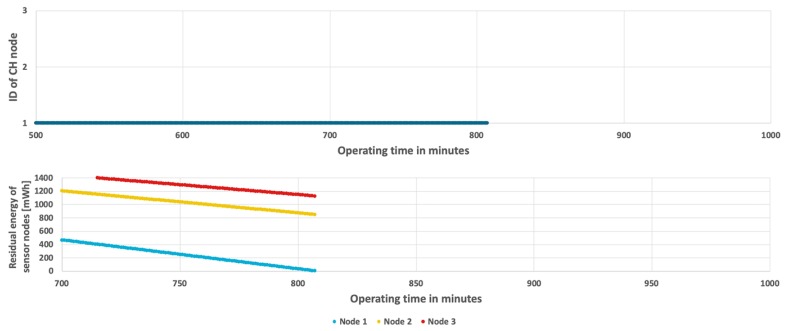
Energy consumption by sensor nodes without CH rotation.

Figure 6 shows the results obtained for the simplest approach, which permanently assigns the CH role to one of the sensor nodes at the initialization stage. The sensor node with ID = 1 acts as CH during the considered period of time, as shown in Figure 6(top). In this case, the CH node (node 1) depletes its battery faster than the cluster members (node 2 and node 3). The WSN lifetime has not exceeded 13 h and 27 min. When the CH node dies, the remaining nodes have over 661 mWh of unused energy (see Figure 6(bottom)). It should be noted that in case of the fixed CH node the same results are observed for both analyzed scenarios (with constant and variable event occurrence probability) because the energy consumption of CH node, which has to send detection results to sink at each step, does not depend on the probability of event occurrence (as presented in Figure 5).

**Figure 7:**
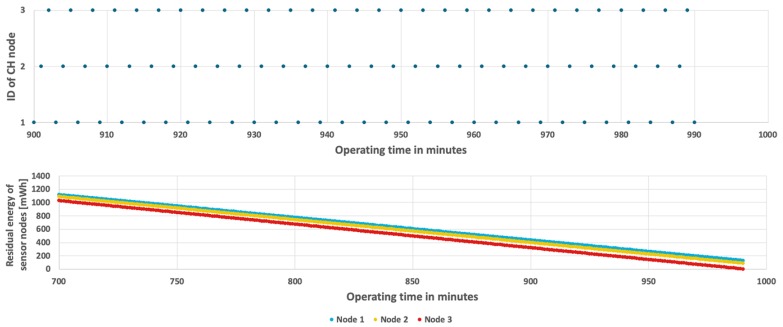
Energy consumption by sensor nodes for RRCR algorithm and fixed probability of event occurrence.

**Figure 8:**
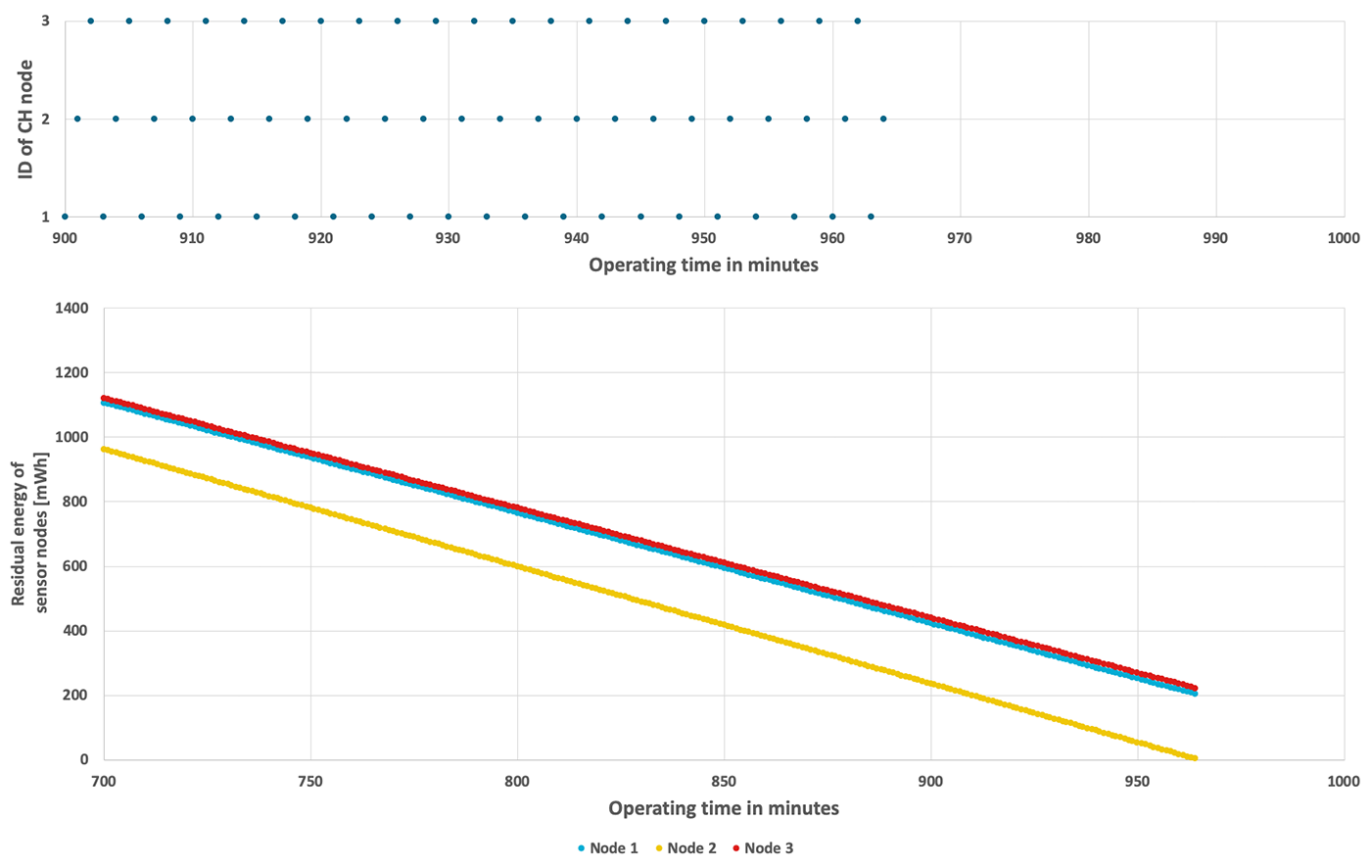
Energy consumption by sensor nodes for RRCR algorithm and variable probability of event occurrence.

The second examined method uses the round-robin CH rotation (RRCR) algorithm [38,39], which is based on the time-driven approach. This algorithm allocates fixed time interval to each sensor node in a cyclic way. Thus, all sensor nodes take the CH role for the same time. During experiments this time interval was set to one minute, based on results of preliminary tests. The WSN lifetime and energy consumption by sensor nodes for the RRCR algorithm are presented in Figure 7 and Figure 8. When comparing these results with those of the fixed CH approach (Figure 6) it is apparent that the introduction of CH rotation significantly extends the network lifetime. In the scenario with fixed probability of event occurrence the WSN lifetime was 16 h 31 min (Figure 7 bottom), while in the second scenario with variable probabilities, the lifetime decreased to 16 h 5 min. It should be noted that at the end of the network operation, two sensor nodes still have 73 mWh and 144 mWh of unused energy on average (Figure 8 bottom). This shows that the energy resources in WSN are used in a not optimal way. The reason lays in the RRCR algorithm, which inherently assumes that the sensor nodes consume energy with the same rate. This assumption is not met in case of the smart sensor nodes that transmit data only when an important event is detected.

**Figure 9:**
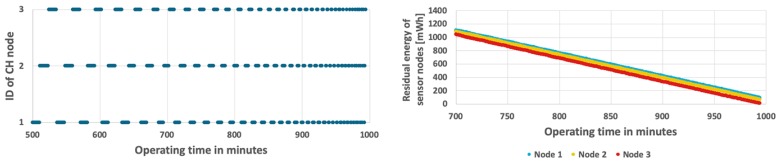
Energy consumption by sensor nodes for EDCR algorithm and fixed probability of event occurrence.

**Figure 10:**
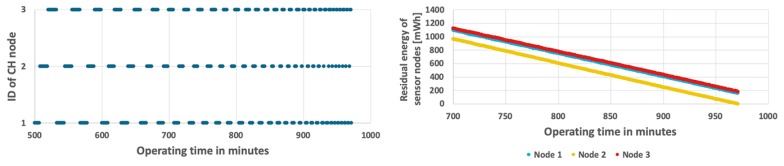
Energy consumption by sensor nodes for EDCR algorithm with a variable number of detected events.

Further improvement of the WSN lifetime was achieved by using the energy-driven CH rotation algorithm (EDCR) [40,41], which takes into account the energy consumption of CH node. According to this algorithm the CH role is moved to the next node in sequence if a given percent of residual energy is consumed by the current CH node. After calibration process, the energy threshold of 5% was used. Results of the EDCR algorithm for fixed and variable event probability are shown in Figure 9 and Figure 10. The left charts in Figure 9 and Figure 10 show changes of CH nodes. It can be observed that the CH changes are performed relatively rarely at the beginning of the network operation, when the residual energy of sensor nodes is high. As energy level decreases, the changes of CH node are made more frequently. In the final stage the change is made every minute. In case when the event occurrence probability is constant, the network lifetime is 16 h and 38 min (Figure 9). As shown in Figure 10, if the event probability is variable, the network lifetime reduces to 16 h and 12 min. The EDCR algorithm adapts CH rotation to the differences of residual energy between particular nodes that are caused by the unequal probabilities of event detection. However, this adaptation is performed with a larger delay, when compared to the proposed approach. The reason is that the decision about CH change in EDCR is taken after the predetermined amount of node’s energy is consumed. In contrast, the proposed method adapts the CH rotation much faster as it directly takes into account the event probabilities and predicts the energy consumption of sensor nodes.

**Figure 11:**
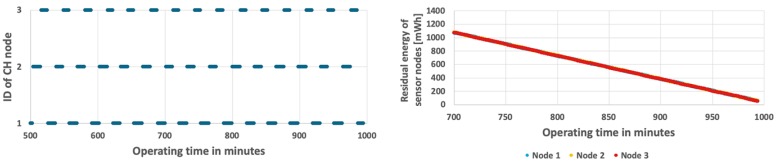
Energy consumption by sensor nodes for the proposed algorithm and fixed probability of event occurrence.

**Figure 12:**
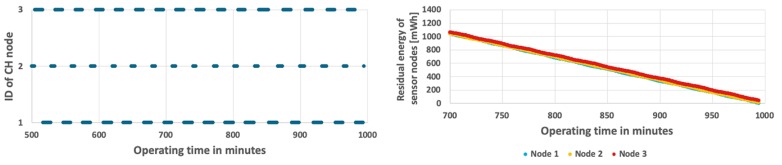
Energy consumption by sensor nodes for the proposed algorithm with a variable number of detected events.

The results achieved for the proposed method are shown in Figure 11 and Figure 12. In case when the probability of event occurrence is constant, the network lifetime was prolonged to 16 h and 48 min (Figure 11). Moreover, the average residual energy at the end of WSN lifetime is only 7 mWh. It means that the energy resources of sensor nodes are almost fully used when using the proposed approach. Figure 12 shows results obtained for the second scenario where probability of event occurrence is changing. In this scenario the WSN lifetime reaches 16 h and 35 min. In the left chart of Figure 12 it is possible to observe length differences of the horizontal bars that depict time intervals in which particular nodes take the CH role. These differences are caused by adaptation of the proposed CH rotation algorithm to currently estimated probabilities of event occurrence for each sensor node. As a result, all sensor nodes consume their energy resources at similar rates. Please note that the sloping lines, depicting energy level in Figure 11 and Figure 12 (right chart) for nodes 1, 2, and 3, are very close to each other. Such observation confirms that the rate of energy consumption by sensor nodes is approximately equal when using the proposed method. The longer WSN lifetime is achieved as the cluster of nodes uses the available energy more effectively in comparison with the state-of-the-art methods. It should be noted that the obtained solution is very close to the optimal one, where all sensor nodes dies exactly at the same time.

**Figure 13:**
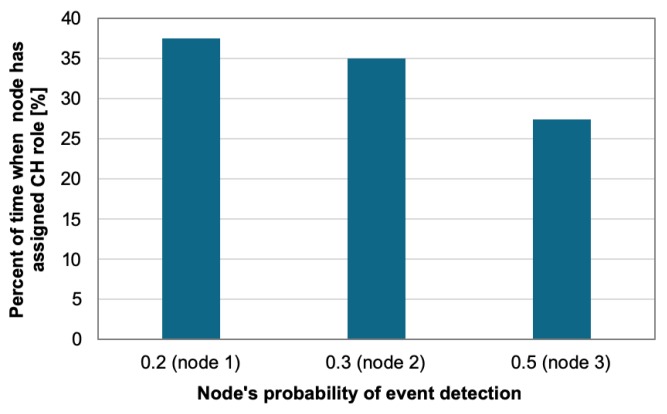
Percent of time when node acts as CH for different probabilities of event occurrence.

Additionally, Figure 13 shows the dependency between the percent of time when node gets CH role and the probability of event detection. These aggregated results relate to the example presented in Figure 11, where each node has fixed event occurrence probability. It can be observed that the more events are detected by a node, the shorter is its time of performing CH role. This leads to balanced energy consumption of the three nodes. In case of node 3, for which the events are detected with the highest probability (0.5), the percent of time when the node acts as CH takes the lowest value (27.5%). In contrast, node 1 has lower probability of event detection than the other nodes (0.3) and takes the CH role for the longest time (37.5%).

**Figure 14:**
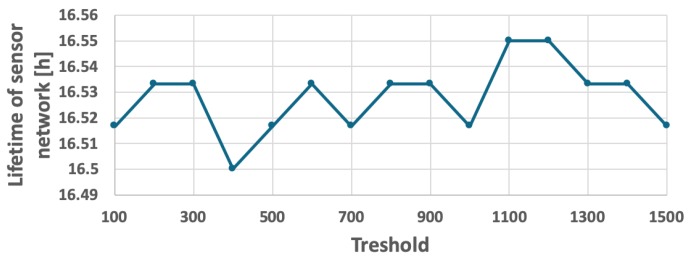
Calibrating the FDCR algorithm for fixed probability of event occurrence.

**Figure 15:**
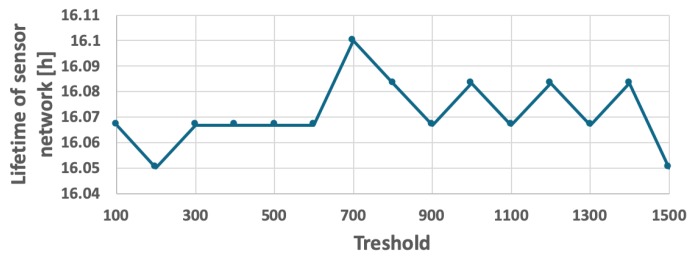
Calibrating the FDCR algorithm for a scenario with a variable number of detected objects.

**Figure 16:**
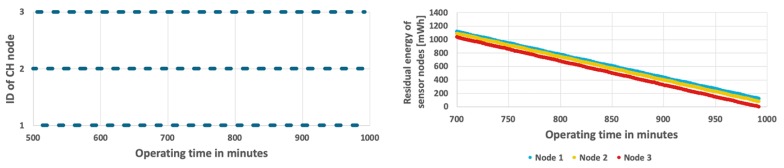
Energy consumption by sensor nodes for FDCR algorithm and fixed probability of event occurrence.

**Figure 17:**
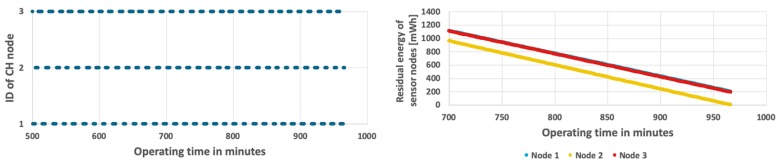
Energy consumption by sensor nodes for FDCR algorithm with a variable number of detected events.

For comparison purposes, another CH rotation method called FDCR (Frame Driven Cluster-head Rotation) was tested. This simple method also takes into account the number of events detected and reported to CH by sensor nodes, but does not involve computations of the CH time limit $TCH_{i}$, thus is slightly easier in implementation. According to this method, the CH role is moved to next sensor node if the number of frames, received by current CH node from cluster members, reaches a predetermined threshold. The threshold of frame number was calibrated independently for both the fixed (Figure 14) and the variable (Figure 15) probability of event detection. Based on the calibration process, the threshold was set to 1100 and 700 frames, respectively. However, results of the experiments in Figure 16 and Figure 17 show that the WSN lifetime for FDCR algorithm is shorter than for the proposed method (16 h 32 min in case of constant event probability, and 16 h 6 min in case of variable event probability). These results are comparable with those of the RRCR algorithm. The lower performance of FDCR is related to the fact that this method ignores the differences between number of events reported by particular sensor nodes.

It should be noted here that the results shown in Figure 6, Figure 7, Figure 8, Figure 9, Figure 10, Figure 11, Figure 12, Figure 13, Figure 14, Figure 15, Figure 16 and Figure 17 were obtained using the data suppression approach to reduce the number of transmissions for each considered method.

Extensive experiments were conducted to compare the above-discussed methods for 10 different scenarios. The experiments involved dynamic scenarios, where the probability of event occurrence changes in time. Each dynamic scenario assumes different changes of the event occurrence probabilities during 15-min cycles, similarly to the second scenario, which is analyzed above in this section. Static scenarios were also included in the experiment. In this case, the event probability for a given node was constant, but different probabilities were assigned to particular nodes.

**Figure 18:**
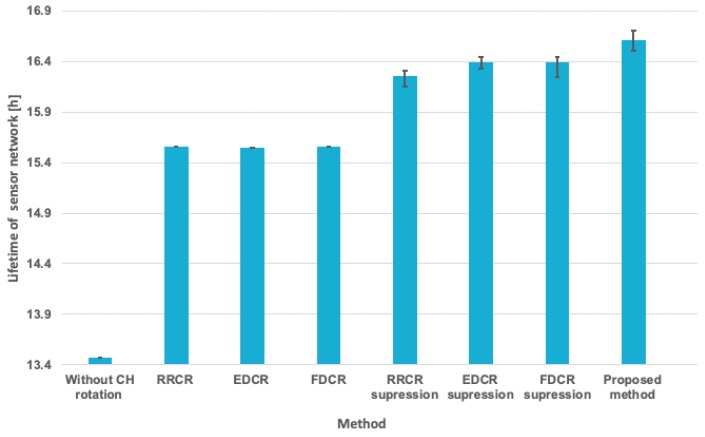
Summarized results of WSN lifetime evaluation for all analyzed scenarios.

The plot in Figure 18 show results of the extended experiments. The columns in this plot correspond to average values of the WSN lifetime for all considered scenarios, while the error bars show maximum and minimum lifetime observed during tests. The average lifetime values in Figure 18 were determined by taking into account the experimental results of the 10 scenarios that were mentioned earlier in this section. Similarly, the maximum and minimum lifetimes were taken from the set of the results obtained for 10 scenarios.

The comparison presented in Figure 18 additionally includes the basic versions of RRCH, EDCR, and FDCR algorithms without suppression of unnecessary transmissions. It can be observed that the suppression approach used by smart sensor nodes greatly contributes to the lifetime extension. At the same time, the proposed method allows us to take full advantage of the smart sensor node capabilities to suppress unnecessary transmissions for prolonging the WSN lifetime.

## 5. Conclusions

The method presented in this paper prolongs the lifetime of WSN with smart sensor nodes. The considered smart sensor nodes have extended computing capabilities and enable comprehensive data processing to transmit information about detected events instead of raw sensor readings. To detect events in larger areas, the smart nodes communicate and cooperate in clusters. One node in cluster must be selected as the CH. The introduced method allows the sensor nodes to decide how long they should take the role of CH to maximize lifetime of WSN. To this end a lightweight energy consumption model is used, which takes into account probabilities of event occurrences for particular nodes. The results obtained during long-term experiments conducted on the hardware testbed shows that the proposed solution gives better results than state-of-the-art approaches when the event probability is constant as well as when the probability changes over time. Thus, it can be concluded that the proposed algorithm enables effective adaptation of the CH rotation process to the current situation of events detection. As for future works, an interesting topic is to examine the CH rotation methods for WSNs with more hierarchy levels and for hybrid sensor nodes that are equipped with different sets of sensors. Other further research directions include evaluation of the method for more complex statistical models with different distributions of event probability and experiments with larger WSNs.
